# Structure and Rheology of Polyelectrolyte Complexes in the Presence of a Hydrogen-Bonded Co-Solvent

**DOI:** 10.3390/polym11061053

**Published:** 2019-06-17

**Authors:** Mor Boas, Gleb Vasilyev, Rita Vilensky, Yachin Cohen, Eyal Zussman

**Affiliations:** 1 NanoEngineering Group, Faculty of Mechanical Engineering, Technion-Israel Institute of Technology, Haifa 32000, Israel; morboas@technion.ac.il (M.B.); mevasil@technion.ac.il (G.V.); rita.vilen@gmail.com (R.V.); 2 Faculty of Chemical Engineering, Technion-Israel Institute of Technology, Haifa 32000, Israel; yachinc@technion.ac.il

**Keywords:** polyelectrolytes, polyelectrolyte complex, co-solvent, SAXS, microstructure

## Abstract

Intermolecular interactions as well as macromolecular conformation affect the rheological and microstructural properties of polyelectrolyte complexes (PECs) solutions. The properties of semi-dilute solutions of weakly charged PECs can be controlled by the degree of ionization and solvent composition. In this work, we examined the effect of ethanol as a co-solvent on PECs composed of poly(allylamine hydrochloride) (PAH) and poly(acrylic acid) (PAA) at low pH. The aqueous PECs solution was turbid, indicating formation of large aggregates, whereas PECs solution in water/ethanol (60:40 *w*/*w*) was transparent, implying no aggregation, and demonstrated higher relative viscosity than the aqueous solution, implying pronounced network formation. Imaging PECs solution by transmission electron microscopy (TEM) demonstrated aggregation, whereas the solution prepared with the mixed solvent revealed almost no phase contrast. Small-angle X-ray scattering (SAXS) of PECs in the aqueous solution indicated the presence of aggregates, while PECs in mixed solvent demonstrated a swelled macromolecular conformation with diminished aggregation. PECs with no ionic interactions in the mixed solvent assumes a homogenous network structure, which enables PECs solution processing by electrospinning.

## 1. Introduction

Processing a polyelectrolyte complexes (PECs) solution is a great challenge due to the strong complexation between the oppositely charged polyelectrolytes and solid formation or aggregation. In general, when both polyelectrolytes are fully charged, there are ionic interactions between the side-groups (PE–PE interactions) as well as hydrogen bonds with the aqueous solvent (PE–solvent interactions) [1,2]. PE-solvent interactions influence chain conformation in a manner that depends on the solvent quality and its dielectric constant [3]. The electrostatic interaction is inversely proportional to the dielectric constant, which is dependent on the solvent composition. For weak polyelectrolytes, the degree of ionization depends on the solvent, whereas for strong polyelectrolytes, addition, for example, of an organic co-solvent, causes counterion-condensation and masking, which lead to shrinking of the fully stretched PE chain [4]. In general, adding an organic hydrogen-bonding solvent may disrupt PE-solvent hydrogen bonding and PE–PE hydrophobic interactions stabilizing electrostatic attraction [5]. When the PE is neutralized, adding a co-solvent increases solvent hydrophobicity, thus increasing solvation [6] and chain swelling [3]. A common way to control the PE–PE interactions is to add salt, which screens and weakens electrostatic interactions between the monomers. Increasing salt concentration in a solution of oppositely charged PEs generally yields structures spanning the range from solid complexes to coacervates (elastic liquid) to dissolved polymer chains [7,8,9].

This work examined the structure and order of semidilute, weakly charged PECs in a mixed solvent. As a model, we used poly(acrylic acid) (PAA) and poly(allylamine hydrochloride) (PAH) dissolved in a mixture of water/ethanol at low pH, where PAA was neutralized and PAH was fully charged. The effects of the addition of a hydrogen-bonding solvent such as ethanol, which may disrupt the hydrogen bonding and hydrophobic interactions stabilizing electrostatic interactions, are discussed. Polyelectrolyte conformation and structure upon complexation were studied via optical transmittance measurements, cryo-transmission electron microscopy (TEM) observations and rheological measurements. The conformation of polyelectrolyte chains in PECs was also examined by small angle x-ray scattering (SAXS). Results demonstrate the significant effect of the co-solvent and the pH on formation of a continuous dense network of PECs.

## 2. Materials and Methods

**Figure 1:**
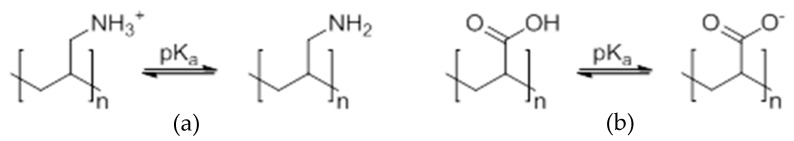
Chemical structure of: (**a**) poly(allylamine hydrochloride) (PAH) and (**b**) poly(acrylic acid) (PAA).

Poly(allylamine hydrochloride) (PAH; *M*_w_: 120,000–180 000 g·mol^−l^; 40% in water; illustrated in Figure 1), was purchased from Polyscience Inc. (Niles, IL, USA), poly(acrylic acid) (PAA; *M*_w_: 450,000 g·mol^−1^; illustrated in Figure 1) and ethanol were purchased from Sigma-Aldrich (St. Louis, MO, USA); all three reagents were used as received. The solutions were prepared with purified water with a resistivity of 18.2 MΩ cm (Bronstead purification system). Next, 10% PAA or PAH were dissolved in water or in a 60:40 (*v*/*v*) pre-prepared water/ethanol mixture and stirred overnight to reach a stable appearance. The PECs solutions were prepared as follows: PAA powder was placed in a glass vial to which the 40% PAH solution was added at a 1:1 molar ratio (by repeating unit). Water/ethanol mixtures were added to the vial to yield 10% (*w*/*w*) solutions having an ethanol content of 0% (aqueous solution), 20%, 40% or 60% (*v*/*v*) and followed by vigorous stirring overnight to reach a stable appearance. A pH-meter reading of 2 (pH 510, Eutech Instruments, Singapore) was adjusted for the prepared solutions using minute quantities of 1M HCl. 

Light transmittance of the solutions was measured with a Genesis10uv (Thermo Scientific, Madison, WI, USA) spectrophotometer at wavelength 700 nm, at 25 °C, equipped with a quartz cell with an optical path of 10 mm.

Discovery DHR-2 rotational rheometer (TA Instruments, New Castle, DE, USA) was used to investigate rheological properties of the solutions under steady-state shear deformation. Cone-plate geometry, with a diameter of 40 mm and an angle at cone tip of 1°, was applied. All tests were carried out at 25 °C. The presented results are the average of three measurements, standard deviation of ±2–5%. 

For cryo-TEM imaging, the specimens were prepared by vitrification according to the protocol reported by Bellare [10]. In order to avoid dissolution of ethanol in ethane, the vitrification of specimens in solutions based on co-solvent was done by plunging into liquid nitrogen. The samples were imaged by a FEI T12 G2 transmission electron microscope operated at 120 kV using the low-dose mode to minimize electron-beam radiation damage. For comparison, 5% solutions of pure components were prepared.

SAXS measurements were performed using a small-angle diffractometer (Molecular Metrology SAXS system, JJ X-Ray A/S, Hoersholm, Denmark), with CuKα radiation from a sealed microfocus tube (MicroMax-002 + S), two Gӧbel mirrors, three-pinholes, and a generator powered at 45 kV and 0.9 mA. The scattering patterns were recorded by a 20 cm × 20 cm two-dimensional position sensitive wire detector positioned 150 cm (SAXS) behind the sample. The resolution of the SAXS system is ~1.16 nm. The scattered intensity *I*(*q*) was recorded in the interval 0.07 < *q* < 2.7 nm^−1^, where *q* is the scattering vector defined as *q* = (4π / λ) sin(θ), 2θ is the scattering angle, and λ is the radiation wavelength (0.1542 nm). Thin-walled quartz capillaries (2 mm in diameter) were filled with the solutions and sealed with epoxy. Measurements were taken at 25 °C, under vacuum. Scattering of the empty capillary and electronic noise were subtracted along with the solvent background. 

The SAXS curve *I*(*q*) of semidilute polymer solutions, is described by the Ornstein-Zernike equation [11]:

(1) $$I\left(q\right)=\;\frac{I\left(0\right)}{1+{\left(q\xi \right)}^{2}}$$

where $I\left(0\right)=\Delta \rho^{2}\varphi^{2}/K_{os}$, φ is the polymer volume fraction, Δρ is the difference in electron density between polymer and solvent, ξ is the correlation length beyond which the inter-segmental excluded volume interactions are screened by the chain overlap, and $K_{os}$ is the osmotic modulus.

To analyze the scattering pattern of an inhomogeneous PECs solution, which is envisioned to be composed mostly of a fluctuating, semi-dilute polymer solution-like structure with embedded molecular aggregates, we added, as was suggested by Hecht et al. [12], the Debye-Bueche equation of an inhomogeneous material to Equation (1):

(2) $$I\left(q\right)=\;\frac{I\left(0\right)}{1+{\left(q\xi \right)}^{2}}+\frac{I_{i}\left(0\right)}{{\left(1+{\left(q\xi_{i}\right)}^{2}\right)}^{2}}$$

where $I_{i}\left(0\right)=8\pi \Delta \rho^{2}\varphi_{i}^{2}\xi_{i}^{3}$ and $\varphi_{i},\;\;\xi_{i}$ represent the volume fraction and characteristic dimension of inhomogeneities, respectively.

## 3. Results and Discussion

### 3.1. Transmittance

**Figure 2:**
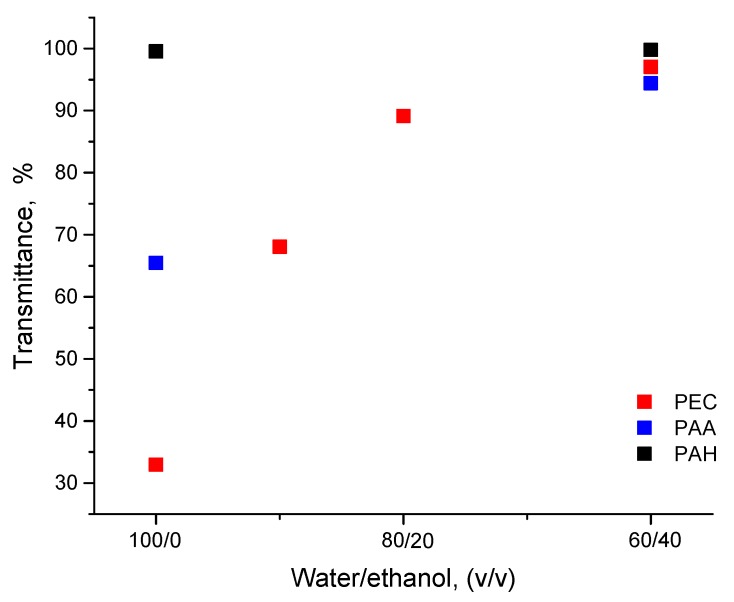
Transmittance of PAA, PAH and PEC solutions with different water/ethanol composition.

The light transmittance of each of the studied polyelectrolytes, i.e., PAA and PAH, and their PECs solutions with different water/ethanol ratios, was measured (Figure 2). The PAA solution demonstrated transmittance of 65% in the aqueous solution, but almost 92% in a 60:40 water/ethanol co-solvent. Almost total light transmittance (~100%) was observed for PAH solutions both in the aqueous solution and a 60:40 water/ethanol co-solvent. Increased transmittance at increased ethanol concentration was measured in PECs solutions of up to 60:40 water/ethanol co-solvent. At higher ethanol concentration the transmittance decreased dramatically (not shown). Low transmittance at 0–10% ethanol resulted in aggregation, while in a solution of 60:40 water/ethanol with nearly total transmittance a homogeneous solution was obtained.

### 3.2. Rheology

The influence of solvent composition on the properties of PECs solutions was further evaluated by measuring the viscosity of the solvents and PECs solutions. Relative viscosity η_r_ = η_0_/η_s_, where η_0_ is the zero-shear rate viscosity of the solution and η_s_ is the viscosity of the pure solvent, was determined to evaluate the contribution of the structure formed by polymer chains to the total viscosity of the solutions (see Table 1). The viscosities of water/ethanol mixtures were well characterized and reached a maximum at 60:40 water/ethanol ratio [13,14]. The relative viscosity of the PECs solution was a function of the water/ethanol ratio. It increased with increasing ethanol content up to 40% but decreased when the ethanol content was 60%. Relative viscosity in a 40% ethanol solvent was two-fold higher than that measured for PAA/PAH in pure water. Considering the transmittance and rheology findings, we chose to further analyze the solution with 60:40 water/ethanol co-solvent.

**Figure 3:**
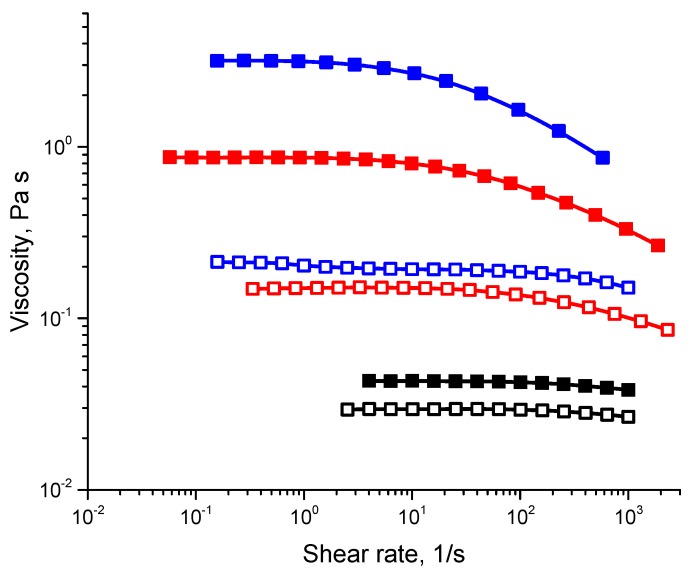
Flow curves of PAA, PAH and PECs in water or water/ethanol co-solvent; black—PAH; blue—PAA; red—PEC; Open squares—dissolved in water; full squares—dissolved in water/ethanol.

Flow curves of PAA, PAH and PECs solutions in water and a 60:40 water/ethanol co-solvent are presented in Figure 3. The 10% PAA solution in water demonstrated close to Newtonian behavior, whereas PAA in water/ethanol exhibited pseudoplastic behavior, since mixing solvents helps to improve the monomer-solvent interactions [6]. The shear viscosity was constant at low stresses and shear thinning was observed at high stresses, a behavior that is typical of most semidilute entangled and concentrated polymer solutions. Pure PAH solutions in both water and water/ethanol demonstrated close to Newtonian behavior implying that the entanglement network was sparse. However, in ethanol/water the viscosity was slightly higher, presumably due to the solvent viscosity. PECs solution in water and water/ethanol exhibited pseudoplastic behavior. However, the PECs solution in water/ethanol demonstrated an approximate 6-fold increase in viscosity versus the aqueous solution, implying a denser network in the solution. It is known that dissolving PAA in a water/ethanol solvent assists in solvation [6], which leads to swelling and an increase in the gyration radius (R_g_) of PAA macromolecules compared to PAA dissolved in water [3,15]. It is likely that this specific co-solvent and its ratio are optimal for PAA swelling [3], with minimal effect on PAH and yielding PECs network formation.

### 3.3. TEM

**Figure 4:**
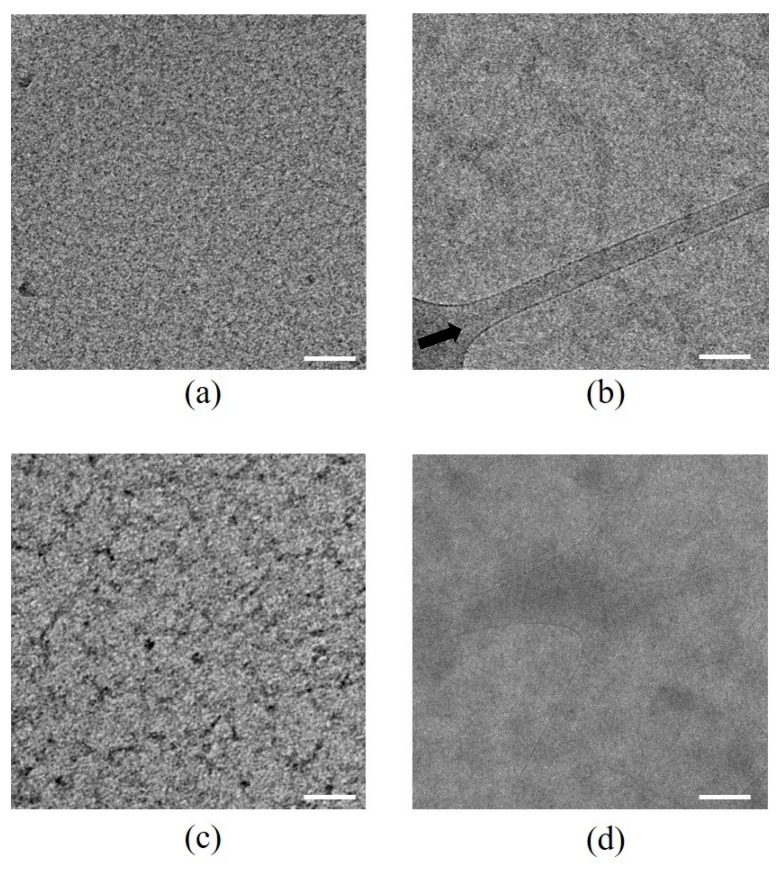
Cryo-TEM images of (**a**) PAA in water (depicting small aggregates), (**b**) PAA in water/ethanol (depicting diminished aggregation, the arrow marks the edge of holes in the supporting carbon film), (**c**) PAA/PAH in water (depicting larger-scale aggregates), and (**d**) PAA/PAH in water/ethanol (depicting a homogeneous solution). Scale bar = 100 nm.

Cryo-TEM images of PAA and PECs in water and water/ethanol co-solvent were examined. Images of PAA in water (Figure 4a) exhibited small spherical aggregates on a length scale of a few nanometers. Dissolving PAA in water/ethanol (Figure 4b) slightly diminished the aggregation and formed a more continuous phase. In the aqueous solution, the neutral PAA chains tended to collapse due to its hydrophobic backbone and self-assemble or aggregate [6,16], while a mixture of water/ethanol broke the hydrophobic PE–PE interactions. PECs in the aqueous solution (Figure 4c) formed branched structures, elongated and entangled, the width of which were approximately 10 nm. Hydrophobic interactions between the polymers seemed to induce self-assembly over dissolution, as observed for PAA in water. This result resembled a previous observation of highly charged, diluted PECs in water, which formed nearly spherical quasi-soluble particles [17]. The structure of PECs in water/ethanol (Figure 4d) was quite different, demonstrating diminished microstructures compared to those that appeared in the aqueous solution, because ethanol molecules, a hydrogen bonding solvent, disrupt PAA-PAH hydrophobic interactions [5]. This solution was characterized by a weak network of elongated macromolecules with intensity differences presumably arising from concentration fluctuation differences, suggesting an almost continuous network of dissolved polymers. 

### 3.4. SAXS

**Figure 5:**
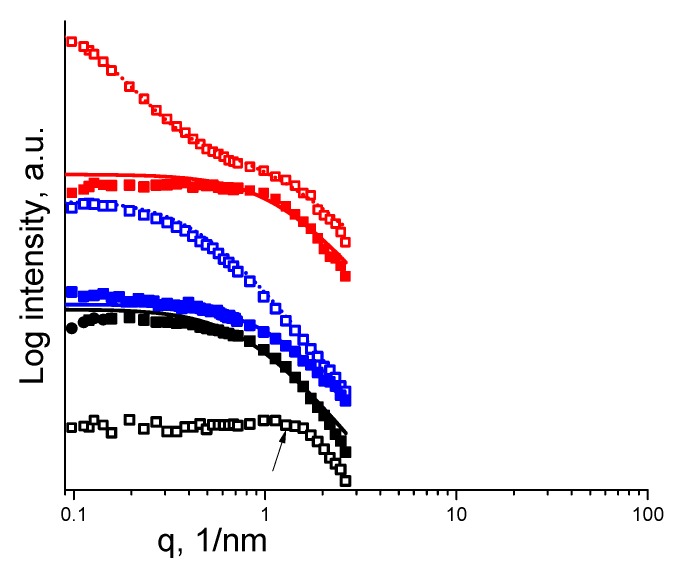
SAXS measurements of PAH, PAA and PECs in water or water/ethanol. PAA and PEC curves are shifted for clarity; black—PAH; blue—PAA; red—PEC; open squares—dissolved in water; full squares—dissolved in water/ethanol. Fitting of the data was performed using Equation (1) or (2). The slopes at a high *q*, were estimated by linear equation. The black arrow marks a broad peak of PAH in water.

The microstructure of the semi-dilute solutions was also characterized using SAXS. Figure 5 shows scattering curves of the pure polymers as well as of the complex solutions in water and water/ethanol co-solvent. The correlation lengths $\xi ,\;\;\xi_{i}$ of the homogeneous phase and the larger inhomogeneities, respectively, are presented in Table 2. The scattering curve of PAA in water decayed at a high *q* range as a power law of *q*^−2^, indicating a random (Gaussian) chain conformation, as exhibited by neutral polymers at theta solvent conditions. PAA in water/ethanol demonstrated a scattering slope of *q*^−1.7^ at a high *q*. The lower fractal dimension of the chains than in the aqueous solution implied the more expanded conformation of a self-avoiding chain in a good solvent. Scattering from the PAH solution in water demonstrated a broad peak at *q* = 2πξ^−1^, typical to curves of polyelectrolytes [8,18]. At a high *q*, the fractal dimension was 2, suggesting adoption of the highly extended directed random walk conformation typical of polyelectrolytes with no salt. The water/ethanol mixture was less influential on PAH than on PAA [19] and was associated with an almost similar slope and fractal dimension. PECs in water demonstrated a distinctive microstructure, with a broad shoulder at intermediate values of the scattering vector *q*, with noticeable excess scattering at low *q* range. This is reminiscent of the patterns that often appear when the shoulder is considered to be due to the fluctuation scattering of the dissolved chains, hence taking on the Lorentzian form (Equation (2)). The excess low-angle scattering is considered to be due to molecular aggregates, exhibiting a fractal dimension of 1.7 [20,21]. PECs in water/ethanol demonstrated a slope of −2 at a high *q* range, implying a random walk conformation of the macromolecules, whereas at a low *q* the excess low-angle scattering is diminished, implying no self-assembly.

## 4. Discussion and Conclusions

**Figure 6:**
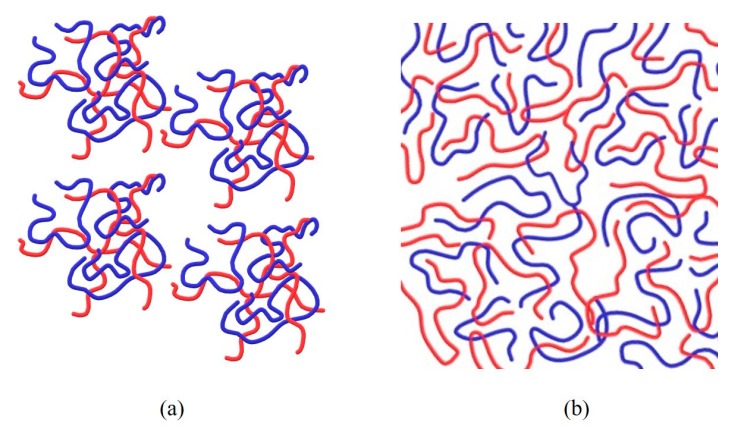
Sketch of PEC’s macromolecular microstructure (**a**) in water and (**b**) in water/ethanol co-solvent. PAH—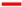, PAA—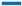.

We have shown that using ethanol as a co-solvent with water for polyelectrolytes composed of poly(allylamine hydrochloride) (PAH) and poly(acrylic acid) (PAA) at low pH can induce formation of a continuous dense network. This was verified by SAXS and rheological and transmittance measurements, which showed no evidence for molecular aggregation, a transparent solution and non-Newtonian behavior [22], suggesting that the complex is soluble but still exhibits a significant degree of complexation (see Figure 6). In contrast, PAA/PAH solutions in the aqueous medium were opaque, with self-assembled aggregates with a diameter lower than 10 nm, demonstrating two length-scales: between chains and between aggregates. It is likely that the water/ethanol co-solvent inhibits aggregation and leads to network formation. The effect of a mixed-solvent system has been suggested to alter the effective solvent affinities to the charged polyelectrolyte, thus assisting formation of a homogeneous solution [23,24]. Therefore, addition of an organic hydrogen-bonding solvent disrupts the hydrogen bonding and hydrophobic interactions stabilizing electrostatic interactions.

The suggested method for polyelectrolyte ordering may facilitate efficient processing of polyelectrolyte complexes. A previous study demonstrated electrospinning of a similar system of polyelectrolytes, which exploited its viscoelastic properties and the existence of a continuous dense network in the pristine solution [25]. Further processing methods should be explored while taking advantage of the pre-ordering of the polyelectrolytes, which may lead to their efficient macroscopic ordering.

## Figures and Tables

**Table 1:** Relative viscosity of PECs in different water/ethanol ratio.

Water/Ethanol	100/0	80/20	60/40	40/60
Relative viscosity	158	169	329	275

**Table 2:** Estimated value of the correlation length according to the solution scattering functions. (Equation (1) or (2)).

Sample Solvent	PAH Water	PAH Water/Ethanol	PAA Water	PAA Water/Ethanol	PECs Water	PECs Water/Ethanol
ξ(nm), ξ_i_(nm)	0.46 *	1.24	2.37	0.73	0.9, 7.8	0.72

* using the peak position marked by the arrow in Figure 5.
